# Post Mortem Findings of Cetaceans Stranded Along the Campania Coast from 2016 to 2022

**DOI:** 10.3390/ani15121812

**Published:** 2025-06-19

**Authors:** Emanuele Esposito, Maria Oliviero, Doriana Iaccarino, Gianluigi Paduano, Francesco Serra, Martina Levante, Maria Grazia Amoroso, Clementina Auriemma, Amalia Gallo, Maria Gabriella Lucibelli, Agata Campione, Roberta Rispoli, Francesca Menafro, Francesca Bove, Maria Dimatteo, Marianna D’Amore, Barbara Degli Uberti, Virginia Mattioda, Federica Giorda, Carla Grattarola, Guido Pietroluongo, Cinzia Centelleghe, Giovanna Fusco, Esterina De Carlo, Fabio Di Nocera

**Affiliations:** 1 Istituto Zooprofilattico Sperimentale del Mezzogiorno, Via della Salute, 2, Portici, 80055 Napoli, Italy; maria.oliviero@izsmportici.it (M.O.); doriana.iaccarino@izsmportici.it (D.I.); gianluigi.paduano@izsmportici.it (G.P.); francesco.serra@izsmportici.it (F.S.); martina.levante@izsmportici.it (M.L.); mariagrazia.amoroso@izsmportici.it (M.G.A.); clementina.auriemma@izsmportici.it (C.A.); amalia.gallo@izsmportici.it (A.G.); mariagabriella.lucibelli@izsmportici.it (M.G.L.); agata.campione@izsmportici.it (A.C.); roberta.rispoli@izsmportici.it (R.R.); francesca.menafro@izsmportici.it (F.M.); francesca.bove@izsmportici.it (F.B.); maria.dimatteo@izsmportici.it (M.D.); marianna.damore@izsmportici.it (M.D.); barbara.degliuberti@izsmportici.it (B.D.U.); giovanna.fusco@izsmportici.it (G.F.); direzionesanitaria@izsmportici.it (E.D.C.); fabio.dinocera@izsmportici.it (F.D.N.); 2 Istituto Zooprofilattico Sperimentale del Piemonte, Liguria e Valle d’Aosta, 10154 Torino, Italy; virginia.mattioda@izsplv.it (V.M.); federica.giorda@izsplv.it (F.G.); 3 Department of Comparative Biomedicine and Food Science, University of Padova, 35020 Legnaro, Italy; guido.pietroluongo@studenti.unipd.it (G.P.); cinzia.centelleghe@unipd.it (C.C.); 4 National Interuniversity Consortium for Marine Sciences (CoNISMa), 00196 Roma, Italy; 5 Interuniversity Center for Cetacean Research (CIRCE), 53100 Siena, Italy

**Keywords:** cetaceans, pathogens, Campania region, Italy, zoonosis, *Cetacean Morbillivirus*, *Brucella ceti*, *Toxoplasma gondii*, animal health

## Abstract

The presence of cetaceans along the Campania coast has always been documented. Between 2016 and 2022, out of 65 cetaceans stranded along the Campania coast, 46 were studied for bacteriological, virological, parasitological, and histopathological investigations. The results highlighted that for 59% (n = 27) of the specimens, the cause of death was of natural origin, while for only 2% (n = 1) of animals, the origin of death was ascribed to anthropic causes. Unfortunately, for 39% (n = 18) of the cetaceans, it was impossible to determine the cause of death. All the cetaceans that died of natural causes showed viral, bacterial, and parasitic infections. The primary pathogens detected were *Cetacean Morbillivirus* (CeMV, 65.2%, n = 30/46), *Toxoplasma gondii* (10.9%, n = 5/46), and *Brucella ceti* (8.7%, n = 4/46). The animals showed typical lesions of the isolated pathogens, such as systemic infection, meningoencephalitis, and pneumonia. Moreover, even with a lower frequency, other relevant pathogens like *Photobacterium damselae*, *Salmonella enteritidis*, *Erysipelothrix rhusiopathiae*, and *Chlamydia abortus* were isolated. These data were useful to understand the spread and circulation of these pathogens, some zoonotic, in the coastal marine waters of the Campania region.

## Simple Summary

In Italy, many studies have been conducted on cetacean diseases and pathogens, but little information is available in the Campania region. This study aimed to describe the post mortem findings of cetacean species stranded along the Campania coast. Forty-six animals were studied between 2016 and 2022, and the leading causes of death were related to infectious origins associated with bacterial, viral, and parasitic pathogens, some of which are also dangerous for human health. The results of this study highlighted that *Cetacean Morbillivirus* was the pathogen that mostly infected cetaceans. This virus may be the main cause of death in these animals, but it can also cause coinfections with other pathogens, such as *Brucella ceti* and *Toxoplasma gondii*. Given the role of marine sentinels and from a One Health perspective, it is important to continue the surveillance and monitoring of cetaceans.

## 1. Introduction

The Mediterranean Sea covers 0.8% of the world’s surface but, despite its small size, it hosts around 17,000 marine species [1]. Among these, the presence of cetaceans along the Campania coast has always been documented [2,3,4,5], with sightings of irregular species in the Mediterranean basin reported in recent years [6]. Cetaceans represent a fundamental component of marine biodiversity; eight species are considered regular in the Mediterranean Sea, such as the striped dolphin (*Stenella coeruleoalba*), the common bottlenose dolphin (*Tursiops truncatus*), the common dolphin (*Delphinus delphis*), the Risso’s dolphin (*Grampus griseus*), the long-finned pilot whale (*Globicephala melas*), the Cuvier’s beaked whale (*Ziphius cavirostris*), the sperm whale (*Physeter macrocephalus*), and the fin whale (*Balaenoptera physalus*) [7].

In the aquatic environment, marine mammals play a prominent role for several reasons, both ecologically as predators at the top of the food web [8] and as sentinels of the health of marine ecosystems [9]. This is due to their longevity, life in a synanthropic habitat, blubber as a reserve of anthropogenic lipophilic toxins [10,11], ability to respond to environmental changes, and being indicators of the human impact on the aquatic ecosystem [12,13,14,15].

Monitoring the mortality of sentinel species such as cetaceans represents a strategic way to assess changes that could concern animal, environmental, and human health from a One Health perspective. To assess the impact of different threats on cetacean health, it is crucial to systematically investigate stranding events through comprehensive post mortem examinations and specific diagnostic investigations, providing valuable insights into the health of the population [16].

Upon completion of the above-mentioned examinations, the pathologist can try to formulate a hypothesis of the cause of death (COD) on the basis of the evidence found. The likely COD can be classified as natural, when related to infectious diseases or non-infectious conditions (i.e., senescence, neonatal/perinatal pathologies, intra or interspecific interactions, degenerative disorders, neoplasias, etc.), and as anthropic, when related to the effects of human activities such as fishery interaction, vessel strikes, etc.

Considering infectious diseases, the presence of emerging diseases may be due to the increase in stressful phenomena, such as environmental degradation, climate change, fishing, noise, and heavy boat traffic, which cause immunosuppression in these animals [17]. Many cases of *Brucella ceti*, *Cetacean Morbillivirus* (CeMV), *Toxoplasma gondii*, and other bacterial, viral, parasitic, and fungal infections have been described in cetacean species [12,18,19,20,21].

Fishing is considered one of the human activities with a significant impact on the aquatic environment, including marine mammals [7,17]. Among the main impacts, by-catch and ingestion are considered the most reported [7], with trawling and longline fishing as the most used gear in the Mediterranean Sea [22].

Collecting samples from stranded cetaceans represents a logistical challenge and the present study summarises data obtained by examining carcasses found stranded along the Campania coast between 2016 and 2022 in an attempt to assess the prevalence of specific pathogens, human interaction, and the likely causes of death (COD).

## 2. Materials and Methods

### 2.1. Post Mortem Examination

Sixty-five stranded cetaceans were reported along the Campania coast between 2016 and 2022 since the local veterinary health institute started its official work on cetacean health and conservation monitoring along the regional coastline.

Campania is an Italian region located in the western Mediterranean Sea, bathed by the Tyrrhenian Sea. The coast has a total length of 500 km, where four gulfs open: Gaeta, Naples, Salerno, and Policastro. The Campania seabed is characterised by the presence of two submarine canyons, the Dohrn Canyon and the Cuma Canyon. The Dohrn Canyon, located about 12 miles from Naples, is one of the deepest in the Mediterranean, reaching 1300 m. The Cuma Canyon, near Ischia, extends up to 800 m deep. The Campania coast is known for being characterised by a high level of coastal urban population, tourism, and commercial fishing [23,24,25].

Forty-six of 65 (70.7%) carcasses were recovered for post mortem investigations [26,27,28,29,30,31,32] according to D.D.G.R.C. 231/2015 [33] and L.R. 7/2023 [34], which include the participation of several public institutes such as research institutions, the regional coast guard, and veterinary health institutes.

The causes of death were hypothesised thanks to the activities of the Istituto Zooprofilattico Sperimentale del Mezzogiorno (IZSM) and the Italian National Reference Centre for Diagnostic Investigations in Stranded Marine Mammals (C.Re.Di.Ma) together with the Department of Comparative Biomedicine and Food Science of the University of Padova.

**Figure 1:**
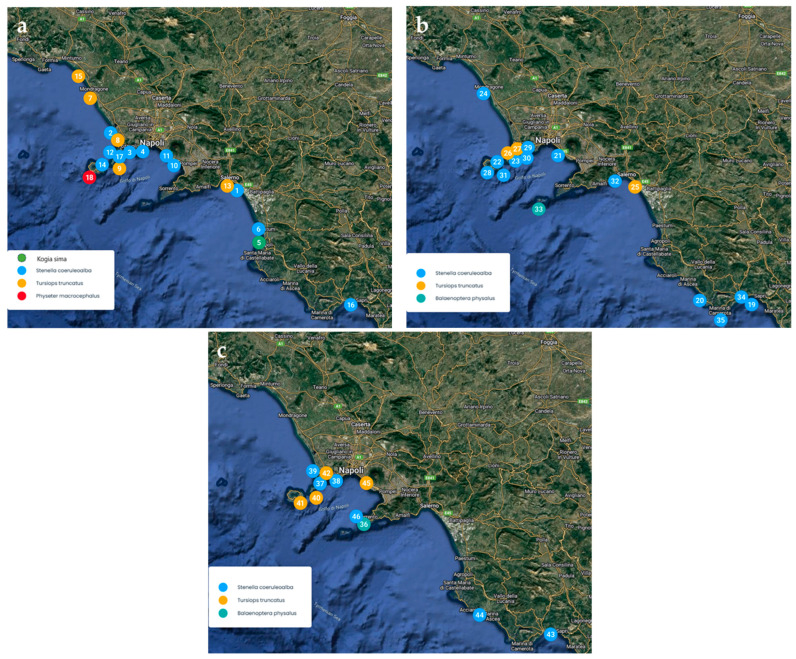
Map of the Campania coast, indicating the stranding locations of the 46 cetacean specimens studied. (**a**) Stranding sites of the triennium 2016–2017–2018. (**b**) Stranding sites of the two-year period 2019–2020. (**c**) Stranding sites of the two-year period 2021–2022.

The geographical distribution of the cetaceans examined during the years studied is represented in Figure 1, which corresponds to the target area of which the local veterinary health institute is in charge. The figure is divided into 3 sub-figures according to the number of strandings for better visual clarity.

All recovered specimens were each registered at the IZSM with a registration code (IZSM code) and at the Banca Dati Spiaggiamenti (BDS) (http://mammiferimarini.unipv.it/, accessed on 3 December 2024) with another identification code.

All 46 carcasses were analysed with a necroscopic examination according to Pugliares et al. [35], Geraci et al. [36], Mazzariol et al. [37], and Ijsseldijk et al. [38]. Several data were collected during the necropsy, including species identification, sex, total body length (TBL) (cm) [38], weight (kg), age class estimated, the decomposition condition category (DCC), nutritional condition category (NCC), and gastric contents, including foreign bodies.

Depending on TBL, and considering the maturation of the gonads, it was possible to establish three estimated age classes: newborn/calf, juvenile, and adult [36,37,39,40,41].

Anatomical indicators, such as the blubber thickness, the rib prominence, and the convexity of the back muscle mass, allowed the evaluation of the NCC and the subsequent classification as good, moderate, and poor [38].

The gastric chambers were opened to evaluate the contents such as food, foreign bodies, and the presence of parasites. The ectoparasites and endoparasites found were collected, washed with physiological saline solution, fixed, and preserved in 70% ethanol for subsequent microscopic/stereoscopic identification, following the morphological keys [42,43,44,45].

During the necropsy, sampling was adapted based on the DCC. For animals classified as extremely fresh or just dead (code 1), fresh (code 2), and with moderate decomposition (code 3), the tissue samples of all major organs were collected and split for various investigations, such as microbiological, biomolecular, and histopathological. Aliquots of some organs were frozen at −20 °C for biomolecular analysis. The Central Nervous System (CNS) aliquots were preserved in 10% buffered formalin for histopathological and immunohistochemical (IHC) investigations and were subsequently sent to C.Re.Di.Ma. For animals classified as in advanced decomposition (code 4) and mummified (code 5), partial investigations such as biomolecular and microbiological were carried out when possible. In some cases, it was possible to collect moderately preserved CNS samples from DCC 4 animals for histopathological investigations.

### 2.2. Microbiology

For general bacteriological investigations, tissue samples, including lymph nodes, liver, spleen, kidney, lung, intestine, and brain, were plated on Blood Agar (BA) and Tryptone Soy Agar with 2% NaCl (TSAS) and subsequently incubated at 37 °C ± 1 °C for 24–72 ± 3 h under aerobic conditions or under microaerophilia conditions in case of granulomatous and abscess lesions. For the intestine samples, plates were incubated under anaerobic conditions. The bacterial colonies grown on the two nutrient media were selected, isolated, and tested with oxidase (Oxoid, Thebarton, South Australia), catalase (bioMérieux, Marcy-l’Etoile, France), a motility test, and Gram stain [46,47]. The different colonies were identified with biochemical micro-method tests (API^®^ 20E and API^®^ 20NE—bioMérieux, Marcy-l’Etoile, France) and miniaturised tests (VITEK2^®^—bioMérieux, Marcy-l’Etoile, France).

Targeted bacteriological assays were performed on target organs to screen for zoonotic pathogens such as *Salmonella* spp., *Listeria monocytogenes*, and *Brucella* spp. For *Salmonella* spp., aliquots of the liver, intestine, and faeces were enriched in Muller Kauffmann Tetrathionate Novobiocin Broth (MKTT) and Rappaport Vassiliadis Soy Broth (RVS). Subsequently, 10 µL of each broth was plated on Xylose Lysine Desoxycholate Agar (XLD) and Brilliance Salmonella Agar (BS), respectively. For *L. monocytogenes*, samples of brain and faeces were pre-enriched in Half Fraser Broth (HF), then enriched in Fraser Broth (FB), and subsequently 10 µL was plated on Listeria Oxford Agar (Oxf).

For *Brucella* spp. detection and identification, target tissue samples were collected and analysed for specific bacteriological procedures in accordance with international recommendations [48]. Suspected colonies of *Brucella* spp. were tested for Gram stain, oxidase, catalase, urease activities, and slide agglutination with polyclonal anti-*Brucella* serum [49] and identified according to Grattarola et al. [50]. Subsequently, colonies of *Brucella* spp. were sent to the National and WOAH Reference Laboratory for Brucellosis, Istituto Zooprofilattico Sperimentale dell’ Abruzzo e del Molise, Teramo, Italy, and identified as *B. ceti* using the PCR-RFLP method [51].

### 2.3. Molecular Investigations

Molecular investigations for detecting CeMV, *Avian Influenza type A Virus* (AIV), *T. gondii*, and *Brucella* spp. were routinely carried out on target organs when possible. Target organs included lymph nodes, liver, spleen, kidney, bladder, lung, heart, and brain for CeMV, lymph nodes, lung, and brain for AIV, muscle, lymph nodes, liver, spleen, kidney, lung, heart, and brain for *T. gondii*, and lymph nodes, liver, spleen, breast, uterus, placenta, testicles, lung, and brain for *Brucella* spp.

Viral detection of CeMV was carried out by using a protocol of real-time RT-PCR capable of identifying a small section of the *Cetacean Morbillivirus* gene F [52].

Molecular detection of AIV was carried out using a QuantStudio 5 Real-Time PCR thermal cycler (Thermo Fisher Scientific, Waltham, MA, USA) and applying a protocol of Real-Time PCR capable of identifying gene M of the *Avian Influenza type A virus* [53].

The Real-Time PCR protocol used for the detection of *T. gondii* was based on targeting the 529 bp repeat element [54]. Real-Time PCR for the detection of *Brucella* spp. was carried out simultaneously by IZSM and C.Re.Di.Ma according to Bounaadja et al. [55] and Grattarola et al. [50], respectively.

Aliquots of various organs such as the skin, lymph nodes, spleen, kidney, lung, and brain were sent to C.Re.Di.Ma for detecting *Herpesvirus* (HV); the analyses were carried out according to VanDevanter et al. [56].

### 2.4. Histopathology and Immunohistochemistry

Histological investigations were performed on representative samples chosen based on organ preservation and carcass decomposition. Tissue samples were collected and fixed in 10% neutral buffered formalin, trimmed, and the sections were embedded in paraffin wax. Then, 3–4 µm thick sections were stained with hematoxylin and eosin (HE) with a standard protocol (Leica Autostainer XL, Nussloch, Germany) and examined under a light microscope.

Neuropathological examination was performed by C.Re.Di.Ma., following the sampling protocol described by Giorda et al. [57].

Immunohistochemistry (IHC) for *Morbillivirus* was routinely performed on tissue sections of all the histologically examined CNS, using a monoclonal anti-canine distemper virus (CDV) antibody (VMRD, Pullman, WA, USA). IHC for *T. gondii* was carried out on CNS sections in cases of microscopic evidence of protozoan infection, using a polyclonal serum of caprine origin (VMRD, Pullman, WA, USA) [58].

### 2.5. Causes of Death Evaluation

The likely COD was hypothesised based on the findings of post mortem investigations and ancillary tests. All data associated with the cases considered were carefully revised, considering previously published data and the availability of diagnostic frameworks recently disclosed, to assess better cetacean post mortem findings, such as LIFE DELFI’s framework for fishery interaction [59] adopted in 2020.

The hypotheses of the COD were classified as either natural, with the origin related to infectious diseases (bacterial, viral, mycotic, parasitic) or non-infectious conditions (i.e., senescence, neonatal/perinatal pathologies, degenerative disorders, etc.) [60,61,62], and as anthropic, when related to an interaction with human activities, with the origin related to fishery interaction, vessel collision, or marine litter ingestion. Regarding fishery interaction, the findings related were categorised as by-catch with active fishing gear, by-catch with passive fishing gear, chronic entanglement, larynx entanglement, ingestion, intentional injury, or by-catch with undetermined fishing gear [59].

## 3. Results

### 3.1. Post Mortem Examination

Forty-six specimens recovered and analysed belonged to five different species of cetaceans: 30 striped dolphins (*S. coeruleoalba*) (65.2%), 12 common bottlenose dolphins (*T. truncatus*) (26%), 1 sperm whale (*P. macrocephalus*) (2.1%), 2 fin whale (*B. physalus*) (4.3%), and 1 dwarf sperm whale (*Kogia sima*) (2.1%) (Table 1).

The sex distribution of the striped dolphins studied included 15 females and 14 males, and it was impossible to identify the sex of 1 specimen. The age categories were as follows: 14/30 (46.7%) adults, 12/30 (40.0%) juveniles/subadults, and 4/30 (13.3%) newborns/calves. Instead, out of 12 bottlenose dolphins, 7 were male, 4 were female, and 1 could not be determined. Similar to the striped dolphins, there was a greater number of adults (8/12; 66.7%) and few specimens belonged to the category of juveniles/subadults (4/12; 33.3%). Of the other cetacean species analysed, the only dwarf sperm whale was an adult female; the only sperm whale was an adult male; finally, both fin whales were female, but one was an adult and the other was a juvenile.

Unfortunately, many carcasses (21/46) were in an advanced decomposition state (DCC 4), 14/46 were fresh (DCC 2), 10/46 were in moderate decomposition (DCC 3), and for a few specimens (1/46), the necropsy examination was limited due to the state of mummification (DCC 5). Furthermore, no animal with DCC 1 was examined.

**Figure 2:**
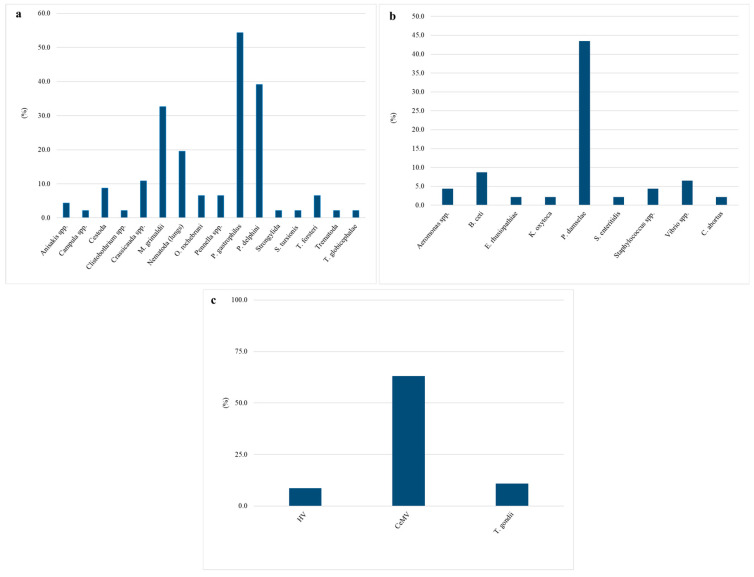
Pathogens isolated and identified during parasitological (**a**), bacteriological (**b**), and molecular (**c**) investigations of the 46 carcasses examined.

The ectoparasites and endoparasites collected during necropsy belonged to various classes, such as nematodes, cestodes, and trematodes. As indicated in Figure 2a, 16 different species were identified; in particular, the most frequent findings were *Pholeter gastrophilus* (25/46; 54.3%), followed by *Phyllobothrium delphini* (18/46; 39.1%) and *Monorygma grimaldii* (15/46; 32.6%).

In carcasses with an advanced state of decomposition (DCC 4 and DCC 5), assessing the lesions was difficult due to organ colliquation and generalised autolysis.

The lesions found and more detailed information such as stranding data, gastric content, and the pathogens and helminths detected are reported for each case in Table S1.

### 3.2. Microbiology

The bacteriological examination was carried out on 45 specimens despite the different conservation codes, while the analysis was not performed on 1 specimen with DCC 5 (ID 28). Of the 45 animals subjected to bacteriological investigation, only in 14 cases bacteria were associated with the COD in the presence of systemic infection and/or the pathological findings potentially associated (ID 5, 10, 15, 16, 22, 29, 31, 32, 34, 35, 37, 38, 43, 44).

As indicated in Figure 2b, *Photobacterium damselae* was more frequently isolated (20/46; 43.5%), followed by *B. ceti* (4/46; 8.7%), *Vibrio* spp. (3/46; 6.5%), *Aeromonas* spp. and *Staphylococcus* spp. (2/46; 4.3%), *Erysipelothrix rhusiopathiae*, *Klebsiella oxytoca*, *Salmonella enteritidis*, and *Chlamydia abortus* (1/46; 2.2%). Several bacteria with potential zoonotic roles were identified, such as *B. ceti*, *E. rhusiopathiae*, *S. enteritidis*, and *C. abortus*.

Specifically, *P. damselae* was associated with systemic infection in one dwarf sperm whale (ID 5) and in seven striped dolphins (ID 16, 22, 29, 32, 34, 35, 43); *B. ceti* was associated with severe non-suppurative meningoencephalitis correlated with plexochoroiditis in three striped dolphins (ID 37, 38, 44); *Vibrio* spp. and *Aeromonas* spp. were detected at a systemic level in two striped dolphins (ID 32, 43), respectively.

*S. enteritidis* and *K. oxytoca* were associated with suppurative pneumonia in a common bottlenose dolphin (ID 15) and pyogranulomatous pneumonia in a striped dolphin (ID 44), respectively. Meanwhile, interstitial pneumonia, disseminated intravascular coagulation, and multisystemic disease were associated with *C. abortus* systemic infection in a striped dolphin (ID 10).

*E. rhusiopathiae* was detected in a mesenteric lymph node of a striped dolphin (ID 12); however, it was not possible to observe the typical lesion due to the decomposition of the tissue.

No positivity to *L. monocytogenes* was found in all specimens analysed. All results are shown in Table S1.

### 3.3. Molecular Investigations

Biomolecular investigations for the detection of CeMV, HV, and *T. gondii* were systematically carried out, and the results highlighted, respectively, 30/46 (65.2%), 4/46 (8.7%), and 5/46 (10.7%) positive specimens, as indicated in Figure 2c.

In particular, CeMV was associated with the COD in 19 cases, in the presence of systemic infections and/or the pathological findings potentially related.

In detail, CeMV was associated with systemic infection in a dwarf sperm whale (ID 5), eight striped dolphins (ID 6, 34, 35, 37, 38, 39, 43, 44), and a common bottlenose dolphin (ID 8). Furthermore, lymphocytic hepatitis associated with *Morbillivirus* systemic infection was observed in a common bottlenose dolphin (ID 7), renal haemorrhages in a striped dolphin (ID 11), encephalitis and umbilical cord infection associated with systemic infection in a striped dolphin (ID 19), cardiac fibrosis in a striped dolphin (ID 20), meningoencephalitis associated with systemic infection in two striped dolphins (ID 21, 24), meningoencephalitis and lymphoid depletion associated with systemic infection in case ID 43, interstitial pneumonia and meningoencephalitis associated with systemic infection in a striped dolphin (ID 22), interstitial pneumonia, lymphocytic epicarditis, and myocarditis associated with systemic infection in case ID 37, reactive lymphadenitis in a striped dolphin (ID 30), and lymphocytic cholangitis associated with systemic infection in a common bottlenose dolphin (ID 42). Instead, in five striped dolphins (ID 12, 23, 29, 32, 46), two common bottlenose dolphins (ID 40, 41), two fin whales (ID 33, 36), and one sperm whale (ID 18), the virus was detected in organs without associated lesions.

Biomolecular evidence of HV was demonstrated in four striped dolphins (ID 29, 37, 38, 43). Specifically, in case ID 29, αHV was detected in the CNS, potentially related to encephalitis. Instead, in case ID 37, αHV was detected in the CNS, in coinfection with *T. gondii* and *Brucella* spp., and in the kidney, and it was considered potentially associated with severe non-suppurative meningoencephalitis and lymphoplasmacellular nephritis.

Furthermore, in cases ID 38 and 43, both αHV and γHV were detected. In case ID 38, bronchointerstitial pneumonia was associated with γHV, whereas αHV was detected in the skin without any associated lesions. In case ID 43, αHV was associated with spleen lymphoid depletion, whereas γHV was detected in lymphnodes without any associated lesions.

Biomolecular evidence of *T. gondii* was demonstrated in four striped dolphins (ID 16, 24, 34, 37) and one common bottlenose dolphin (ID 45). In particular, in cases ID 16, 24, and 37, the protozoan was detected in the CNS causing non-suppurative meningoencephalitis. Instead, in ID 34 *T. gondii* was associated with a systemic infection, particularly in the CNS, causing pyogranulomatous meningoencephalitis.

Finally, in case ID 45, *T. gondii* was detected only in the muscle without any associated lesions.

All results are shown in Table S1.

### 3.4. Histological and Immunohistochemical Investigations

Despite the different DCCs, histopathological investigations were carried out on 41/46 (89.1%) carcasses. All organs were analysed in 32/41 (78.0%) cases, while only some organs were examined in 9/41 (22.0%) cases. Instead, no samples were collected for four common bottlenose dolphins (ID 25, 26, 40, 41) and one striped dolphin (ID 28) due to general tissue degradation.

Significant histopathological findings were found in the organs of all systems. Specifically, in the integumentary system, severe pyogranulomatous dermatitis and panniculitis of unknown origin were reported in a striped dolphin (ID 31).

In the lymphatic system, the main histopathological findings were eosinophilic lymphadenitis in two common bottlenose dolphins (ID 9, 42) and five striped dolphins (ID 22, 23, 30, 35, 44), and splenitis in two striped dolphins (ID 23, 38) associated with bacterial and viral infection. In addition, a striped dolphin (ID 11), a sperm whale (ID 18), and a common bottlenose dolphin (ID 42) reported lymphoid hyperplasia, thrombosis, and generalised atrophy at the splenic level, possibly associated with CeMV infection. Lastly, splenic lymphoid depletion was noted in a striped dolphin (ID 43), perhaps related to α-*Herpesvirus* infection.

**Figure 3:**
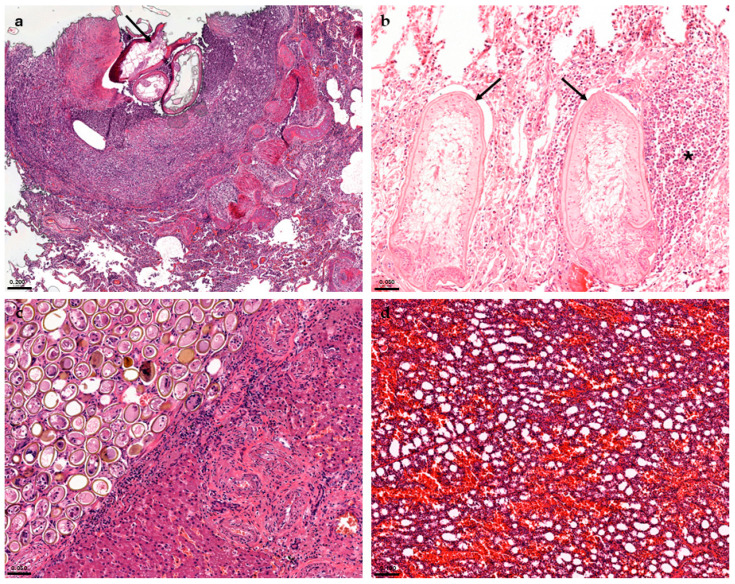
(**a**) Parasitic pneumonia (ID 23). Granuloma characterised by central sections of adult nematodes (arrow) surrounded by inflammatory cells and fibroblasts. H&E (4x). (**b**) Parasitic enteritis (ID 21). Mucosal granuloma (asterisk) characterised by epithelioid macrophages, neutrophilic granulocytes, and numerous eosinophils surrounding sections of adult nematodes (arrows). H&E (20x). (**c**) Parasitic hepatitis (ID 44). Nodule delimited by a thin capsule of fibrous connective tissue containing operculate eggs and trematode larvae. H&E (20x). (**d**) Parasitic mastitis (ID 35). Diffuse interstitial inflammatory infiltrates in the mammary gland. H&E. (10x).

In the respiratory system, bronchopneumonia in striped dolphins (ID 19, 21, 22, 23, 32, 34) and pneumonia in common bottlenose and striped dolphins (ID 8, 10, 31, 35, 37, 38) of parasitic (Figure 3a), bacterial, and viral aetiology were reported. In addition, pulmonary atelectasis and pleuritis were detected in cases ID 18 and 32, respectively, probably due to infectious origin.

Regarding the cardiovascular system, cardiac intravascular and subendocardial thrombosis were reported in cases ID 18 and 43, respectively. Degenerative myocardial lesions associated with *C. abortus* and CeMV, respectively, were highlighted in cases ID 10 and 44. Finally, CeMV infection was associated with fibrosis and inflammatory processes of epicardium and myocardium in cases ID 20 and 37.

In the digestive system, enteritis was found only in striped dolphins (ID 11, 21, 22, 35, 37, 38, 44), two of which were of parasitic origin (Figure 3b). In addition, parasitic gastritis was reported in two common bottlenose dolphins (ID 8, 9) and in seven striped dolphins (ID 23, 24, 34, 37, 38, 39, 44). Various histopathological findings were reported in the liver; in particular, hepatitis, cholangitis, and cholangiohepatitis were found both in common bottlenose and in striped dolphins (ID 7, 11, 19, 20, 23, 35, 37, 42, 43, and 44) (Figure 3c). Lastly, degenerative phenomena were highlighted in cases ID 10, 32, and 43.

About the urinary system, renal congestion and lymphoplasmacellular nephritis were reported in two striped dolphins (ID 11, 37), associated with CeMV and α-*Herpesvirus* infection, respectively.

**Figure 4:**
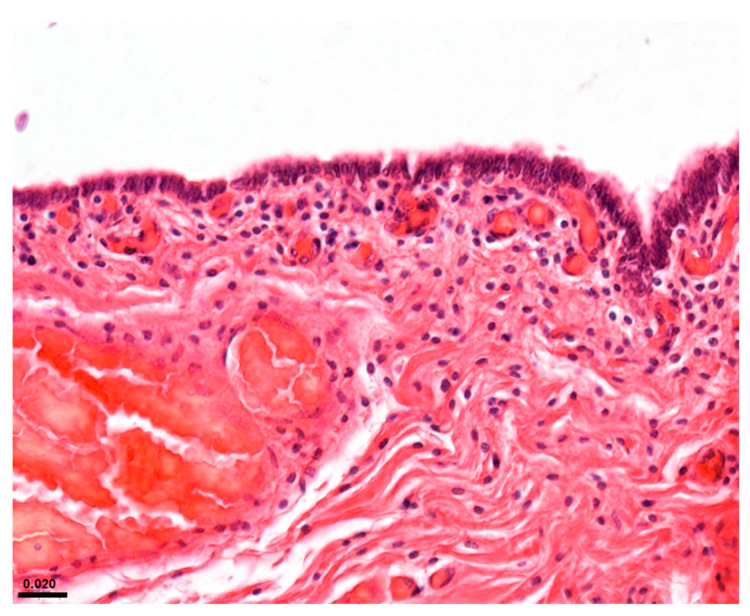
Endometritis (ID 38). Uterus characterised by moderate subepithelial inflammatory infiltrates (lymphocytes) and numerous vascular thrombi. H&E (40x).

Considering the reproductive system, cases ID 35 and 38 showed parasitic mastitis (Figure 3d) and endometritis (Figure 4), respectively. Instead, case ID 43 showed lymphocytic endometritis and ovaritis possibly associated with infectious origin.

**Figure 5:**
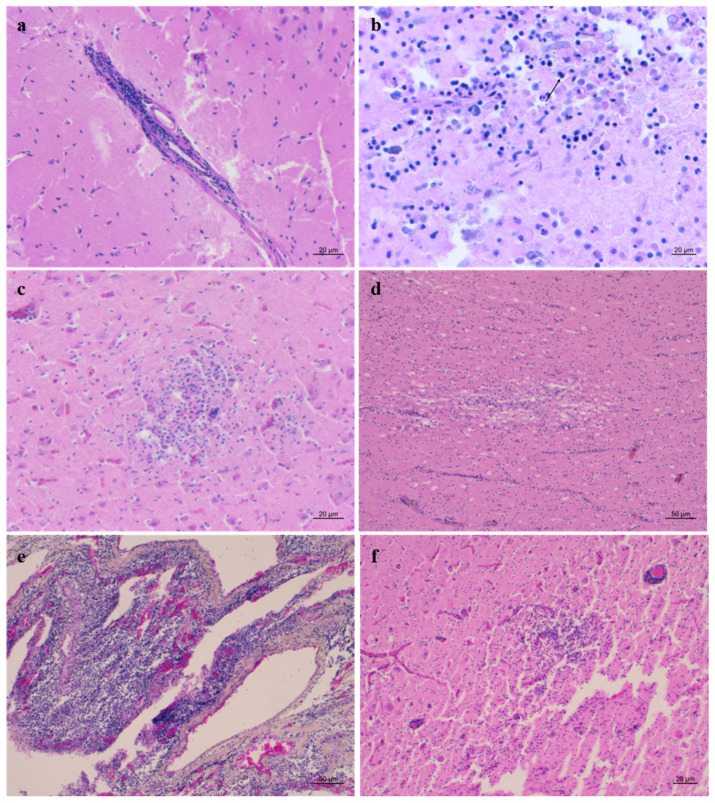
(**a**) Frontal cortex (ID 24). Mild non-suppurative (NS) meningitis. H&E (10x). (**b**) Cortex (ID 34). Severe malacic area with several gitter cells and few syncytia cells (arrow). H&E (20x). (**c**) Cortex (ID 21). Focus of gliosis and neuronophagia. H&E (10x). (**d**) Cortex (ID 37). Area of moderate spongliosis in white matter. H&E (10x). (**e**) Plexus choroideus (ID 37). Severe and widespread NS plexus choroiditis. H&E (10x). (**f**) Thalamus (ID 37). Severe granulomatous encephalitis with perivascular cuffings and numerous protozoan cysts. H&E (10x).

In conclusion, for the nervous system, the histopathological findings highlighted encephalitis associated with CeMV infection in three striped dolphins (ID 19, 21, 34). Moreover, in a striped dolphin (ID 16), granulomatous meningoencephalitis was related to *T. gondii* infection, and in cases ID 22, 24, and 43, non-suppurative meningoencephalitis was compatible with CeMV infection. Furthermore, in ID cases 37 and 38, non-suppurative meningoencephalitis and plexochoroiditis related to coinfection with *B. ceti*, *T. gondii*, and CeMV were observed. Neuropathological pictures were selected and are shown for some cases in Figure 5.

Immunohistochemistry investigations were performed on the CNS of five striped dolphins (ID 16, 21, 24, 34, 37). In particular, cases ID 16 and 37 showed positivity for *T. gondii* antigens, while positive results for CeMV antigens were reported for cases ID 21 and 24. Finally, positivity for *T. gondii* and CeMV antigens was found only for case ID 34.

### 3.5. Causes of Death Evaluation

**Figure 6:**
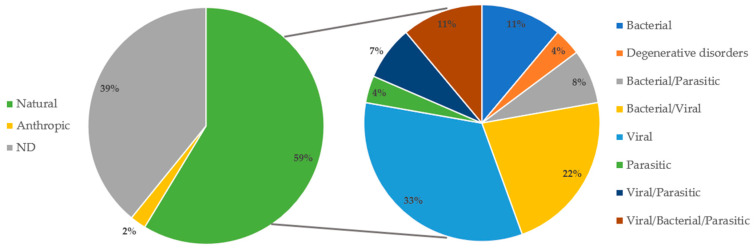
On the (**left**) are the classifications of COD categories (natural, anthropic, and ND) in percentages. On the (**right**) are the COD sub-categories (bacterial, degenerative disorders, bacterial/parasitic, bacterial/viral, viral, parasitic, viral/parasitic, viral/bacterial/parasitic) in percentages.

The likely COD was hypothesised for 28 specimens out of 46 examined (cases ID 5, 6, 7, 8, 9, 11, 13, 15, 16, 19, 20, 21, 22, 23, 24, 29, 30, 31, 32, 34, 35, 37, 38, 39, 42, 43, 44, 45), of which 59.0% (27/46) was classified as natural and 2.0% (1/46) as anthropic; while for the remaining 18 specimens (cases ID 1, 2, 3, 4, 12, 14, 17, 18, 25, 26, 27, 28, 33, 36, 40, 41, 45, 46) (39.0%; 18/46), the COD remained undetermined (ND), as shown in Figure 6.

Natural COD with the origin related to infectious diseases was the most represented (26/27; 96.3%), with only one case associated with non-infectious conditions (1/27; 3.7%).

Natural COD with the origin related to infectious disease was hypothesised in the presence of systemic infections and/or pathological findings potentially related to the specific agent in one or more organs, as specifically shown in Table S1.

In detail, a COD related to viral infections was reported in 33.0% (9/27) of cases (six striped dolphins and three common bottlenose dolphins) (ID 6, 7, 8, 11, 19, 20, 21, 39, 42), specifically to CeMV infection. A COD associated with bacterial infections was reported in 11.0% (3/27) of cases (two striped dolphins and one common bottlenose dolphin) (ID 10, 15, 32). In particular, case ID 10 reported a systemic infection by *C. abortus*, case ID 15 was characterised by suppurative pneumonia associated with *S. enteritidis* infection, and case ID 32 reported systemic infections due to *P. damselae* and *Vibrio* spp. A COD associated with parasitic infections was reported in a striped dolphin (4.0%; 1/27) (case ID 23) showing severe parasitic bronchopneumonia.

For 13 specimens (48.0%; 13/27) the COD was related to coinfections, and the viral/bacterial were the most represented (22.0%; 6/27) (cases ID 5, 22, 29, 38, 43, 44). Specifically, case ID 5 was characterised by a systemic infection by CeMV and *P. damselae*, case ID 22 by meningoencephalitis and interstitial pneumonia associated with CeMV systemic infection and *P. damselae* systemic infection, case ID 29 by encephalitis associated with *Herpesvirus* infection and *P. damselae* systemic infection, case ID 38 by CeMV systemic infection, bronchointerstitial pneumonia by γHV, and non-suppurative meningoencephalitis associated with *B. ceti*, case ID 43 by non-suppurative meningoencephalitis and lymphoid depletion associated with CeMV systemic infection, lymphoid depletion perhaps also associated with αHV, and *P. damselae* systemic infection, and case ID 44 by CeMV systemic infection, non-suppurative meningoencephalitis associated with *B. ceti*, and pyogranulomatous pneumonia associated with *K. oxitoca*.

Moreover, viral/bacterial/parasitic coinfections were highlighted in three striped dolphins (11.0%; 3/27) (ID 34, 35, 37). In particular, case ID 34 was characterised by pyogranulomatous meningoencephalitis related to systemic infection by CeMV and *T. gondii* and *P. damselae* systemic infection, while case ID 35 showed CeMV and *P. damselae* systemic infections, associated with multiorganic parasitosis, and case ID 37 presented a non-suppurative meningoencephalitis associated with *Herpesvirus*, *B. ceti*, and *T. gondii*.

Finally, viral/parasitic coinfections were detected in two striped dolphins (7.0%; 2/27) (ID 24, 30), with non-suppurative meningoencephalitis associated with CeMV and *T. gondii* infection and reactive lymphadenitis associated with CeMV infection and multiorganic parasitosis, respectively. Parasitic/bacterial coinfections were also detected in 2/27 cases (7.0%), a common bottlenose dolphin (ID 15) and a striped dolphin (ID 31), with granulomatous meningoencephalitis associated with *T. gondii* infection and *P. damselae* systemic infection, and parasitic pneumonia with unknown origin systemic neutrophilic inflammation, respectively.

**Figure 7:**
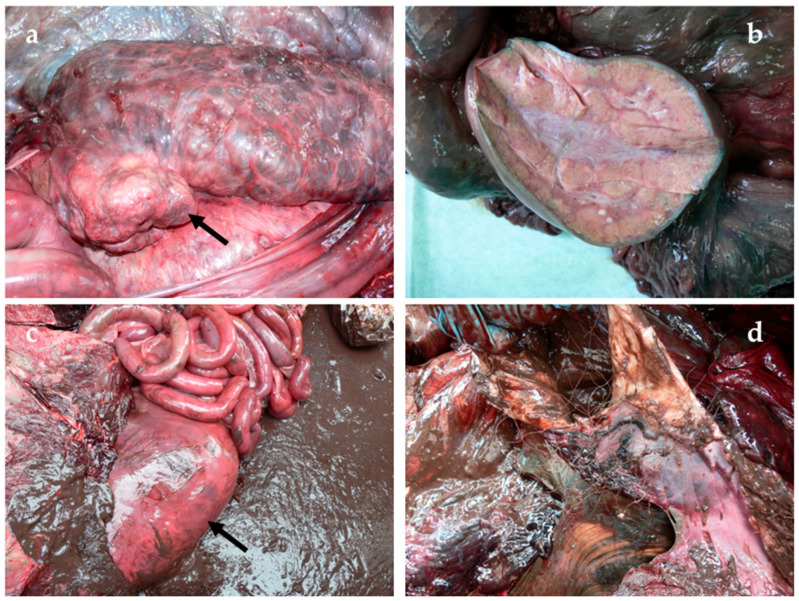
(**a**) Calcified nodular renal mass (187 gr) found during necropsy (arrow). (**b**) Nodular renal mass in the section. (**c**) Stomach filled with undigested material before opening (arrow). (**d**) Upon opening, the stomach was filled with a monofilament net, which caused obstruction.

For one common bottlenose dolphin (case ID 9) (4.0%; 1/27), the natural code related to non-infectious disease was associated with degenerative disorders. Specifically, granulomatous hepatitis, with haemorrhages and calcified nodules, calcified nodular renal mass (Figure 7a,b), and necrotic intussusception of the proximal intestine were observed.

The only anthropic COD concerned an adult male bottlenose dolphin (case ID 13) that was suffocated by a monofilament net, about 15 m long, found in the pharynx, glottis, oesophagus, stomach, and intestine, causing their obstruction (Figure 7c,d).

These results are shown in Table S1.

## 4. Discussion

The results obtained, following post mortem examinations and diagnostic investigations, contribute to improving the knowledge of different factors (natural and anthropic) involving the stranding of cetacean species along the coast of Campania.

Of the 65 cetaceans stranded dead in the period between 2016 and 2022 along the Campania coast, 46 specimens (70.7%) were subjected to a post mortem examination, a higher percentage than other national and international studies [60,61,63,64,65]. Following the microbiology, molecular, histological, and immunohistochemical investigations, the COD was hypothesised for 28 animals (61.0%; 28/46), in line with the national percentage [61,63] but with a lower percentage than other European studies [62,65,66].

Unfortunately, for the remaining 18 specimens (39.0%; 18/46), the COD remained undetermined due to excessive decomposition of the animals and the absence of signs related to anthropic impact or injuries attributable to pathogenic agents. This data represents a lower percentage than the Canary Islands [60] and the Catalonian [65] studies; however, it is similar compared to other national data [61,67].

The natural COD was attributed to 27 cases, of which 26 were associated with an infectious disease, in the sub-categories of bacterial, viral, and parasitic, and one was attributed to a non-infectious condition. These data confirm that natural causes of death are the most common in the Mediterranean Sea [60,61,62,65].

Instead, anthropic COD was confirmed in only one case and was associated with fishery interaction, in the sub-category entanglement and ingestion, which represent the most common and documented anthropogenic activities globally [68,69,70]. The low percentage of anthropic COD is in line with other studies [60,61,62,67].

Moreover, although the advanced state of decomposition did not allow the determination of the COD, in some cases (ID 1, 3, 4, 12, 14, 18, 28, 33, 36, 40, 41, 45, 46) the exposure of these animals to various pathogens was detected, such as *P. damselae*, *Vibrio* spp., *E. rhusiopathiae*, CeMV, and *T. gondii*.

Considering the main pathogens detected in the animals studied, CeMV was identified in 30 animals (65.2%; 30/46) and the COD in nine cases. This data emphasised a high presence of this viral agent in the waters of the Campania region, in accordance with Vargas-Castro et al. [71]. CeMV is an RNA virus belonging to the *Morbillivirus* genus and is responsible for mortality events that endanger cetaceans’ health and conservation [72,73,74]. CeMV disease can present in acute, subacute, and chronic forms [75]; the associated lesions are various, such as viral encephalitis and bronchopneumonia/bronchiolointerstitial, which frequently affects both lungs and general immunosuppression [76]. CeMV was detected both as a systemic infection and in single organs. Pathological findings detected in the carcasses were reactive lymphadenitis, lymphocytic cholangitis and hepatitis, cardiac fibrosis, interstitial pneumonia and bronchopneumonia, and NS meningoencephalitis [57,71,77,78,79].

Another pathogen detected with a high percentage (43.5%; 20/46) was *P. damselae*, related to the COD in eight cases together with other pathogens. This bacterium is normally present in aquatic environments and is considered a zoonotic pathogen; in humans it can cause opportunistic infections and necrotising fasciitis [80,81,82,83,84]. *P. damselae* was isolated in several aquatic animals, including farmed fish [85,86,87], sharks [88,89], molluscs [90,91], sea turtles [92,93,94,95], and various cetacean species [72,80,96,97]. The large literature reports the presence of the bacterium in different cetacean species [98,99,100], as an opportunistic agent in cases of unusual mortality events [72]. It was demonstrated that *P. damselae* can cause widespread haemorrhages, ulcers [98], and pneumonia [100] in cetaceans, confirming the lesions found in the animals of this study. Indeed, in this study, the lesions associated with *P. damselae* were systemic infections and multifocal haemorrhages.

Even with low percentages, zoonotic pathogens, such as *T. gondii* (11.0%; 5/46), *B. ceti* (8.7%; 4/46), *E. rhusiopathiae* (2.2%; 1/46), and *C. abortus* (2.2%; 1/46) were detected in the cetaceans studied.

Specifically, *T. gondii* is a worldwide distributed protozoan capable of infecting various animals, including humans. Nevertheless, human infection occurs through the consumption of undercooked meat containing cysts or food or water contaminated with oocysts shed from cats [101]. In cetaceans, *T. gondii* can cause various lesions such as NS meningoencephalitis, disseminated parasitaemia, necrotising placentitis, abortion, necrotising and haemorrhagic pneumonia, lymphadenitis, hepatitis, and adrenalitis [77].

The presence of this parasite in the marine environment is probably due to coastal anthropogenic pressure, flood events that favour the spread of terrestrial pathogens, and the resistance of oocysts to survive even in seawater [102]. For cetaceans from offshore areas, direct contact with contaminated wastewater from ships and the presence of vectors, such as rodents and cats, have been hypothesised as an additional source of transmission [103].

In this study, this protozoan was associated with the COD in five cases, together with other pathogens, of which four were striped dolphins and one was a bottlenose dolphin. The four cases of striped dolphins showed systemic infection, granulomatous, and NS meningoencephalitis, which is in accordance with what is reported in the literature [58,104,105]. The only positivity in *T. truncatus* was in the muscle with no associated lesions.

*Brucella* spp. are zoonotic bacteria that affect domestic, terrestrial [106,107], and aquatic animals, in particular cetaceans and pinnipeds [19,50,108,109,110,111], with chronic diseases [106,107,108,111,112]. These bacteria can survive for a long period of time in the environment and multiplies within the hosts. The effects of *Brucella* infection are similar in humans and animals. Human infection occurs through direct contact with infected tissue or blood and contaminated food. Instead, the transmission of the bacteria in marine mammals can occur through sexual intercourse and breastfeeding with infected hosts or through the placenta from the mother to the foetus [113].

Initially, these bacteria are localised in the lymph nodes; however, if they are present within the blood, they replicate and can spread to various tissues, especially the reproductive tract [112]. Within the *Brucella* genus, *B. ceti* and *B. pinnipedialis* infect marine mammals. *B. ceti* infections are highlighted in the Mediterranean Sea and worldwide [50,110,113,114,115,116]; this bacterium is associated with various lesions such as meningoencephalitis [109,117,118,119,120], abortion, disco spondylitis, subcutaneous abscesses, and endometritis [113]. Actually, in this study *B. ceti*, in coinfection with other viral and parasitic pathogens, was associated with the COD of three cases. The animals examined and positive for this bacterium presented typical lesions such as non-suppurative meningoencephalitis.

Instead, *E. rhusiopathiae* is a zoonotic and ubiquitous bacterium, able to survive long periods of time in the environment, and has been isolated in aquatic and terrestrial animals [121,122,123,124,125,126]. This bacterium is known for swine erysipelas, a disease of great economic importance that affects pigs. This infection affects various vertebrate and invertebrate animals, such as pigs, sheep, horses, chickens, seals, sea lions, cetaceans, mink, squirrels, crustaceans, and freshwater and saltwater fish [127]. Similarly to *B. ceti*, human infection occurs through contact with infected animals, contaminated products, water, waste, or soil. The pathogenesis and transmission route of *E. rhusiopathiae* in marine mammals is still poorly understood. Transmission from terrestrial to aquatic ecosystems has been hypothesised, which has also occurred for *T. gondii* [128]. The disease manifests similar effects in animals and humans; in the former, skin infections and polyarthritis are highlighted [129]; while in humans, it manifests as skin lesions, septicaemia, and endocarditis [130]. *E. rhusiopathiae* infections in cetaceans are widely documented [131,132,133,134] and have been found in several species, such as *Stenella frontalis* and *T. truncatus* [128,135,136,137], *Phocoena phocoena* and *Phoca vitulina* [138], and *Delphinus delphis* [139]. Furthermore, this bacterium causes two forms of disease in cetaceans: an acute fatal septicaemic form and a milder cutaneous form [140]. In this report, *E. rhusiopathiae* was isolated in the mesenteric lymph nodes of an adult male striped dolphin, showing parasitic bronchopneumonia and coinfection between the bacterium of our interest and CeMV, found in the kidney.

Finally, *C. abortus* is a zoonotic bacterium belonging to the *Chlamydiaceae* family. It is primarily considered a pathogen of goats and sheep, causing abortion. This bacterium also affects other mammals, such as cattle, pigs, and horses, including humans [141]. The transmission of *C. abortus* occurs through contact with vaginal fluids and placental membranes at the time of abortion or childbirth or by the inhalation of aerosols from the environment [142]. Despite the importance of these bacteria, there are no data on the pathogenesis of *Chlamydiaceae* in aquatic animals, although cases of their exposure to these pathogens were reported. The transmission route of these bacteria is still unknown; however, as with other pathogens, the spread from terrestrial animals to the aquatic environment can be hypothesised [143].

In this study, *C. abortus* was associated with COD in one case. The pathological findings found in this specimen were interstitial pneumonia, disseminated intravascular coagulation, and multisystemic disease.

In conclusion, the only case of human interaction identified was related to fishery interaction, specifically to entanglement and ingestion, and associated with COD. This finding suggests that this species is more exposed to this interaction due to its habitat and behaviour with human activities [22]. Due to the advanced state of decomposition, no other lesions were evaluated, and it was not possible to detect the presence of bacterial, viral, and parasitic pathogens.

The results of this study highlighted that these animals can be infected by various pathogens with a risk to their short and long-term conservation, many of which are zoonotic and dangerous for human health. Since the Mediterranean Sea is a semi-enclosed basin, marine mammals that inhabit these waters are vulnerable to human activities, such as fishing, noise pollution, maritime traffic, industrial activities, and the presence of pathogens [102,144]. Climate change is weakening the immune responses of cetacean populations, increasing stress, and facilitating the introduction of new pathogens. Exposure to coastal species and sharing common habitats makes the transmission of zoonotic infections from dolphins to humans likely [16].

Unfortunately, despite continuous progress in diagnostic protocols, cases with undetermined causes of death are still present with a high percentage. All this suggests the need for the continuous monitoring of these marine mammals for health surveillance purposes, improvement of the regional stranding network in terms of animal recovery speed, interaction between the different partners of the local stranding network, and harmonisation of data analysis.

## 5. Conclusions

This study provides data on the health status and the pathogens circulating among cetaceans in the waters of Campania.

The data obtained may be useful to improve standardised diagnostic approaches and increase knowledge of the pathogenetic role of the microorganisms that infect these animals.

The results showed the presence in marine environments of several zoonotic pathogens, underlining the importance of marine organisms and habitats as reservoirs of pathogens capable of potentially affecting human health. Finally, fishery interaction was the only reported human activity threatening cetacean conservation in this area. Given the role of marine sentinels and from a One Health perspective, it is important to continue the surveillance and monitoring of cetaceans.

## Figures and Tables

**Table 1:** Stranded cetaceans recovered and analysed during the studied period.

Year	*S. coeruleoalba*	*T. truncatus*	*P. macrocephalus*	*B. physalus*	*K. sima*	Total Strandings per Year
2016	4	0	0	0	0	4
2017	2	3	0	0	1	6
2018	5	2	1	0	0	8
2019	8	3	0	0	0	11
2020	5	0	0	1	0	6
2021	5	3	0	1	0	9
2022	1	1	0	0	0	2
Total	30	12	1	2	1	46

## Data Availability

The data presented in this study are available within the article and Supplementary Materials.

## Supplementary Materials

The following supporting information can be downloaded at https://www.mdpi.com/article/10.3390/ani15121812/s1, Table S1: Examined cetaceans stranded along the coast of the Campania Region between 2016 and 2022.
