# Supplementary material for: Post Mortem Findings of Cetaceans Stranded Along the Campania Coast from 2016 to 2022
Source: Animals (Basel). 2025 Jun 19;15(12):1812. doi: 10.3390/ani15121812 (PMC12189110; doi:10.3390/ani15121812)
Supplement: Supplementary file 1 [file animals-15-01812-s001.zip › animals-3591905-supplementary.pdf]

Table S1. Examined cetaceans stranded along the coasts of the Campania Region between 2016 and 2022. For each detailed information is reported: IZSM identification code, date and location of stranding, species, sex, estimated age class, DCC, NCC, gastric content/presence of marine litter, main macro and microscopic findings, pathogens and helminths detected, etiological diagnosis (relevant pathological findings), classification of the cause of death (COD), origin, sub-category, evidence of fishery interaction (category, description, rate). References of previous papers reporting some of the selected cases are also detailed.

Case ID	IZSM code	Stranding Date	Stranding Location	Species	Sex	Estimated Age Class	DCC	NCC	Gastric content/ marine litter	Main macro and microscopic findings*	Pathogens and Helminths Detected**	Etiological diagnosis (relevant pathological findings)	COD	Origin	Sub-category	Fishery interaction	Category	Description	Rate	Ref.
1	4414	10/01/2016	Salerno (SA)	Striped dolphin	M	Newborn	3	Poor	Absent/-	**Macro:** multifocal haemorrhages (lung, MES ln., meninges); splenic hypertrophy; parasitic nodular granulomatous gastritis	*P. damselae* (intestine)	Multifocal haemorrhages	ND							[61]
2	28026	09/02/2016	Napoli (NA)	Striped dolphin	F	Newborn	2	Poor	Absent/-	**Macro:** splenic hypertrophy and congestion; multifocal haemorrhages (blubber, LNs, liver, vagina, intestine, lungs, CNS); hyperplasia of LNs, tonsil, spleen; **parasitic nodular granulomatous gastritis**; pericarditis and valvular endocarditis.	*Crassicauda* spp., *M. grimaldii*, *P. delphini* (muscle); ***P. gastrophilus*, *O. rochebruni*** (stomach); *T. globicephalae* (intestine)	Multifocal haemorrhages; multiorganic parasitosis	ND							[61]
3	31398	10/02/2016	Napoli (NA)	Striped dolphin	M	Juvenile	4	Moderate	Absent/-	**Macro:** emphysema and haemorrhagic pneumonia; **parasitic nodular granulomatous gastritis**; mesenteric hemorrhagic lymphadenopathy; valvular endocarditis.	*Vibrio* spp. (CNS); *Crassicauda* spp., *M. grimaldii*, *P. delphini* (muscle); ***P. gastrophilus*, *O. rochebruni*** (stomach)	Multiorganic parasitosis	ND							[61]
4	42380	06/03/2016	Posillipo (Na)	Striped dolphin	M	Juvenile	4	Poor	Absent/-	**Macro:** hemoperitoneum; haemorrhages (MES ln., pancreas, lungs and meninges); **parasitic nodular gastritis**	*P. damselae* (intestine); *Crassicauda* spp. (muscle); ***P. gastrophilus*** (stomach); *T. forsteri* (intestine); *P. delphini* (subcutis)	Multiorganic parasitosis	ND							[61]
5	21089	04/02/2017	Agropoli (Sa)	Dwarf Sperm Whale	F	ND	3	Poor	Present/-	**Macro:** intramuscular abscess; hemoperitoneum; haemorrhagic tracheobronchitis; haemorrhagic necrotic pneumonia; haemorrhagic nephritis; valvular endocarditis; cerebral haemorrhage.	**CeMV** (liver, spleen, lung, heart, intestine); *Penicillum* spp., *Geotrichum* spp. (lung); ***P. damselae*** (liver, spleen, PSC ln., MES ln., kidney, synovial	*Morbillivirus* systemic infection; *P. damselae* systemic infection	Natural	Infectious	Viral Bacterial					[61]

Case ID	IZSM code	Stranding Date	Stranding Location	Species	Sex	Estimated Age Class	DCC	NCC	Gastric content/ marine litter	Main macro and microscopic findings*	Pathogens and Helminths Detected**	Etiological diagnosis (relevant pathological findings)	COD	Origin	Sub-category	Fishery interaction	Category	Description	Rate	Ref.
											fluid); *P. delphini* (blubber); *Anisakis* spp. (stomach, intestine)									
6	40071	08/03/2017	Capaccio (Sa)	Striped dolphin	M	Adult	3	Poor	Absent/-	**Macro:** severe haemorrhagic pneumonia; mediastinal lymphadenopathy; splenic hypertrophy; haemorrhagic enteritis; fibrinous peritonitis; **parasitic nodular gastritis;** hyperemic meninges.	**CeMV** (lung, spleen, liver, heart, kidney, LNs); ***P. gastrophilus*** (stomach); *Anisakis* spp.(intestine); *O. rochebruni* (liver and pancreas); Cestoda (muscle)	*Morbillivirus* systemic infection; multiorganic parasitosis	Natural	Infectious	Viral					[61]
7	61249	05/05/2017	Mondragone (Ce)	Common bottlenose dolphin	F	Adult	4	Poor	Absent/-	**Macro:** splenic congestion; haemothorax, hemopericardium; haemorrhagic nephritis; mesenteric caseous lymphadenopathy; **parasitic nodular gastritis**. **Micro: lymphocytic hepatitis**	**CeMV** (liver, lung, CNS); ***P. gastrophilus*** (stomach)	Lymphocytic hepatitis associated to *Morbillivirus* systemic infection	Natural	Infectious	Viral					[61]
8	84719	10/07/2017	Napoli	Common bottlenose dolphin	F	Adult	4	Moderate	Scarce/-	**Macro:** generalized autholysis; **prescapular lymphadenopathy**; **parasitic nodular gastritis;** haemorrhagic pneumonia; cerebral colliquation. **Micro: parasitic gastritis**; parasitic granulomatous pneumonia	**CeMV** (liver, heart, kidney, PSC ln., PUL ln., MES ln., CNS); *P. damselae* (intestine); ***P. gastrophilus*** (stomach)	*Morbillivirus* systemic infection	Natural	Infectious	Viral					[61]
9	87761	18/07/2017	Procida (Na)	Common bottlenose dolphin	M	Adult	3	Moderate	Present/-	**Macro:** haemothorax; pulmonary edema and congestion; granulomatous hepatitis, with haemorrhages and calcified nodules; necrotic intussusception of the proximal intestine; calcified nodular renal mass (187 gr); mesenteric lymphadenopathy. **Micro:** mesenteric eosinophilic limphadenitis	*P. damselae* (liver, kidney, lung, MES ln.); Nematoda (lung); *S. tursionis* (intestine)	Degenerative disorders; intussusception	Natural	DD						[61]
10	141463	04/12/2017	Torre Annunziata (Na)	Striped dolphin	F	Juvenile	2	ND	ND	**Macro:** liver congestion; splenic hypoplasia, with capsular petechiae;	***C. abortus*** (spleen, liver, heart, lung); *Staphilococcus*	Interstitial pneumonia, disseminated intravascular	Natural	Infectious	Bacterial					[61,140]

Case ID	IZSM code	Stranding Date	Stranding Location	Species	Sex	Estimated Age Class	DCC	NCC	Gastric content/ marine litter	Main macro and microscopic findings*	Pathogens and Helminths Detected**	Etiological diagnosis (relevant pathological findings)	COD	Origin	Sub-category	Fishery interaction	Category	Description	Rate	Ref.
										**parasitic gastritis**; pulmonary congestion with irregular pleural surfaces, consolidation, and fibrosis; hyperemic meninges. **Micro: disseminated intravascular coagulation (DIC); interstitial pneumonia**; hepatocyte degeneration; acute myocardial degeneration; **multiorganic cytoplasmic inclusions consistent with *Chlamydia* spp** (lung, heart, liver, prescapular lymphnode, liver, spleen)	spp. (kidney); *P. damselae* (skin); *Aspergillus* spp. (lung); *Pennella* spp. (skin); *Crassicauda* spp. (uterus); ***P. gastrophilus*** (stomach); *M. grimaldii* (peritoneum); *P. delphini* (blubber); *Tetrabothrius forsteri* (intestine)	coagulation and multisystemic disease associated to *C. abortus* systemic infection; multiorganic parasitosis								
11	9793	17/01/2018	Torre del Greco (Na)	Striped dolphin	F	Juvenile	2	Good	Present/-	**Macro:** sero-sanguinous fluid in the thorax and abdomen; splenic hypertrophy; **parasitic nodular gastritis;** diffuse hemorrhages in the intestinal serosa; **renal hemorrhages**; emphysema and haemorrhagic pneumonia; valvular endocarditis; hyperemic meninges and brain. **Micro:** lymphoid hyperplasia (spleen, LNs); cholangitis; haemorrhagic enteritis; **renal congestion**; encephalitis	**CeMV** (kidney); *P. damselae* (lung); ***P. gastrophilus*** (stomach)	Renal haemorrhages associated to *Morbillivirus* infection; encephalitis of unknown origin; multiorganic hemorrhagic lesions (intestine, lung, meninges and brain; *P. damselae* infection	Natural	Infectious	Viral					[61]
12	31518	27/02/2018	Pozzuoli (Na)	Striped dolphin	M	Adult	4	Poor	Absent/-	**Macro:** generalized autolysis; parasitic **nodular granulomatous gastritis**; fibrinous peritonitis; parasitic bronchopneumonia	*E. rhusiopathiae* (MES ln.); CeMV (kidney); *P. delphini* (blubber); ***P. gastrophilus*** (stomach); *T. forsteri* (intestine); Nematoda (lung)	*E. rhusiopathiae* and *Morbillivirus* infection; fibrinous peritonitis of unknown origin	ND							[61]
13	36045	09/03/2018	Salerno (Sa)	Common bottlenose dolphin	M	Adult	4	Poor	Absent/ Net (esophagu	**Macro:** generalized autolysis; multifocal subcutaneous and		Larynx entanglement (setnet); fishing gear (setnet) obstruction	Anthropic	Fishery interaction	Larinx entanglement, ingestion	X	Larinx entanglement, ingestion	Fishing gear (15 m setnet)	C	[61]

Case ID	IZSM code	Stranding Date	Stranding Location	Species	Sex	Estimated Age Class	DCC	NCC	Gastric content/ marine litter	Main macro and microscopic findings*	Pathogens and Helminths Detected**	Etiological diagnosis (relevant pathological findings)	COD	Origin	Sub-category	Fishery interaction	Category	Description	Rate	Ref.
									s, stomach, intestine)	muscular necrotic areas; setnet (15 m) entangling the larynx, obstruction in esophagous, stomach and intestine		in esophagus, stomach, intestine; absence of recently ingested gastric content						obstuctive at larinx, oesophageal, gastric and intestinal level		
14	38911	16/03/2018	Ischia (Na)	Striped dolphin	F	Juvenile	4	Poor	Absent/-	**Macro:** generalized autolysis; splenic hypertrophy; haemorrhagic and parasitic pneumonia; urinary bladder haemorrhagic suffusions; hyperemic meninges	Nematoda (lung)		ND							[61]
15	83037	20/07/2018	Sessa Aurunca (Ce)	Common bottlenose dolphin	F	Adult	4	Poor	Absent/-	**Macro:** generalized autolysis; prescapular lymphadenopathy; **pulmonary abscess;** peritoneal adhesions.	***S. enteritidis*** **(**intestine, lung); *P. damselae* (lung); *P. gastrophilus* (stomach)	Suppurative pneumonia associated to *S. enteritidis* infection	Natural	Infectious	Bacterial					[61]
16	117850	30/10/2018	Ispani (Sa)	Striped dolphin	M	Adult	3	Good	Absent/-	**Macro**: hepatic congestion; splenic petechial haemorrhages; **parasitic nodular granulomatous gastritis;** haemorrhagic enteritis; emphysema and haemorrhagic pneumonia. **Micro: granulomatous meningoencephalitis**	***T. gondii*** (CNS; IHC +); ***P. damselae*** (CNS, lung, liver, kidney, spleen, MES ln, intestine) *P. delphini* (blubber); ***P. gastrophilus*** (stomach)	Granulomatous meningoencephalitis associated to *T. gondii* infection; *Photobacterium damselae* (systemic infection)	Natural	Infectious	Parasitic Bacterial					[61]
17	137488	10/12/2018	Bacoli (Na)	Striped dolphin	M	Adult	4	Poor	Absent/-	**Macro:** generalized autolysis; splenic petechial haemorrhages; parasitic nodular granulomatous gastritis; urinary bladder haemorrhagic suffusions; cerebral colliquation	*P. delphini* (subcutis)		ND							[61]
18	4418	24/12/2018	Forio d'Ischia (Na)	Sperm whale	M	Adult	4	ND	Present/ Plastic fragments (stomach)	**Macro:** generalized autolysis; pharyngeal lesions. **Micro:** thrombosis and generalized splenic atrophy; pulmonary atelectasis; cardiac intravascular thrombosis	CeMV (spleen); *Aspergillus* spp., *Tricophyton* spp., *P. damselae* (pharynx)	*Morbillivirus* infection	ND							[61]
19	16054	27/01/2019	Ispani (Sa)	Striped dolphin	F	Adult (Pregnant)	2	Good	Scarce/-	**Macro:** generalized congestion; parasitic bronchopneumonia; **parasitic nodular granulomatous gastritis;** splenic hypertrophy. **Micro:** parasitic cholangitis;	**CeMV** (CNS, lung, heart, liver, spleen, kidney, MES ln, umbilical cord); *P. delphini* (subcutis,	Encephalitis and umbilical cord infection associated to *Morbillivirus* systemic infection (vertical transmission)	Natural	Infectious	Viral					[54,61,68]

Case ID	IZSM code	Stranding Date	Stranding Location	Species	Sex	Estimated Age Class	DCC	NCC	Gastric content/ marine litter	Main macro and microscopic findings*	Pathogens and Helminths Detected**	Etiological diagnosis (relevant pathological findings)	COD	Origin	Sub-category	Fishery interaction	Category	Description	Rate	Ref.
										**parasitic gastritis**; parasitic bronchopneumonia; **encephalitis (fetus: pneumonia)**	muscle); nematodes (lung); ***P. gastrophilus*** (stomach); Cestoda (bile ducts)									
20	22309	06/02/2019	Centola (Sa)	Striped dolphin	M	Juvenile	4	Good	Absent/-	**Macro:** generalized congestion (liver, kidney, lung); hepatic hypertrophy; splenic hypertrophy; haemorrhagic gastritis; hyperaemic meninges and brain. **Micro:** hepatic perivascular and periductal cuffs of inflammatory cells; **subepicardial and subendocardial fibrosis**	**CeMV** (spleen, heart)	Cardiac fibrosis associated to *Morbillivirus* infection	Natural	Infectious	Viral					[61]
21	23881	08/02/2019	Torre del Greco (Na)	Striped dolphin	M	Adult	3	Poor	Absent/-	**Macro**: haemorrhagic pneumonia**;** parasitic bronchopneumonia; **parasitic nodular gastritis;** urinary bladder haemorrhagic suffusions**. Micro: non-suppurative encephalitis;** parasitic bronchopneumonia; parasitic enteritis	**CeMV** (CNS, MES ln, lung, heart, liver, spleen, kidney, bladder; IHC+ CNS); ***P. gastrophilus*** (stomach) Nematoda (lung)	Meningoencephalitis associated to *Morbillivirus* systemic infection	Natural	Infectious	Viral					[54,61,68]
22	25702	12/02/2019	Porto d'Ischia (Na)	Striped dolphin	M	Juvenile	2	Good	Absent/-	**Macro:** liver congestion; **parasitic nodular granulomatous gastritis;** haemorrhagic enteritis; haemorrhagic pneumonia; hyperaemic brain. **Micro: non-suppurative meningoencephalitis; interstitial bronchopneumonia**; exosinophilic enteritis and lymphadenitis	**CeMV** (CNS, MES ln, lung, heart, liver, spleen, kidney, bladder, PSC ln); ***P. damselae*** (CNS, liver, MES ln)); ***P. gastrophilus*** (stomach)	Interstitial pneumonia and meningoencephalitis associated to *Morbillivirus* systemic infection; *P. damselae* systemic infection	Natural	Infectious	Viral Bacterial					[61]
23	42310	26/03/2019	Procida (Na)	Striped dolphin	F	Juvenile	2	Good	Absent/-	**Macro:** mesenteric lymphadenopathy; splenic hypertrophy; **parasitic nodular granulomatous gastritis**; intestinal haemorrhages; renal haemorrhages; pulmonary congestion and **severe parasitic bronchopneumonia by nematodes**;	**CeMV** (PSC ln.); ***P. gastrophilus*** (stomach); **Nematoda** (lung)	*Morbillivirus* infection; severe parasitic bronchopneumonia	Natural	Infectious	Parasitic					[61]

Case ID	IZSM code	Stranding Date	Stranding Location	Species	Sex	Estimated Age Class	DCC	NCC	Gastric content/ marine litter	Main macro and microscopic findings*	Pathogens and Helminths Detected**	Etiological diagnosis (relevant pathological findings)	COD	Origin	Sub-category	Fishery interaction	Category	Description	Rate	Ref.
										hyperaemic meninges and brain. **Micro: parasitic gastritis** and cholangiohepatitis; eosinophilic lymphadenitis; congestion and lymphoplasmacellular splenitis										
24	44648	03/04/2019	Mondragone (Ce)	Striped dolphin	F	Adult	3	Good	Absent/ Plastic and rope (stomach)	**Macro:** haemorrhagic gastritis, cystitis and metritis; haemorrhagic pneumonia; hyperaemic meninges and brain**. Micro: non-suppurative meningoencephalitis**	**CeMV** (CNS, lung, liver, bladder, MES, PSC an PUL lns; IHC+ CNS); ***T. gondii*** (CNS); *P. delphini* (blubber)	Non-suppurative meningoencephalitis associated to *Morbillivirus* systemic infection; *T. gondii* infection	Natural	Infectious	Viral Parasitic					[54,61]
25	71311	18/06/2019	Pontecagnano Faiano (Sa)	Common bottlenose dolphin	M	Juvenile	4	Good	Absent/-	**Macro:** generalized autolysis			ND							[61]
26	74824	28/06/2019	Procida (Na)	Common bottlenose dolphin	M	Adult	4	Good	Present/ Net esophagus	**Macro:** presence of setnet in the oral and esophageal lumen; generalized autolysis			ND			X	ingestion	fishing gear (set net) in the mouth and in esophagus; presence of recently ingested gastric content	C	[61]
27	88087	13/08/2019	Torregaveta (Na)	Common bottlenose dolphin	M	Adult	4	Poor	Absent/-	**Macro:** monofilament net in the oral cavity, retained in the teeth (presumably post-mortem relief); generalized autolysis			ND							[61]
28	128659	28/11/2019	Forio d'Ischia (Na)	Striped dolphin	ND	Newborn	5	Poor	ND	**Macro:** generalized autolysis; cerebral colliquation	CeMV (CNS)	*Morbillivirus* infection	ND							[61]
29	141936	23/12/2019	Pozzuoli (Na)	Striped dolphin	M	Juvenile	2	Poor	Absent/-	**Macro:** generalized congestion; pulmonar multifocal blackish spots; parasitic pneumonia; hepatomegaly; splenomegaly; **parasitic gastritis**, hepatitis and enteritis; renal haemorrhages; meningeal and cerebral congestion. **Micro: focal non-suppurative encephalitis**, with severe neuronal necrosis and neuronophagia, associated to mild pyogranulomatous plexochoroiditis	***α-herpesvirus*** (CNS); **CeMV** (lung, heart); ***P. damselae*** (CNS, lung, testicle, liver, kidney, spleen, PSC and MES lns); *M. grimaldii* (muscle); Strongylidae spp. (lung, intestine); Cestodea (liver, intestine); ***P. gastrophilus*** (stomach)	Encephalitis associated to *Herpesvirus* infection; *Morbillivirus* infection; *P. damselae* systemic infection	Natural	Infectious	Viral Bacterial					[61]

Case ID	IZSM code	Stranding Date	Stranding Location	Species	Sex	Estimated Age Class	DCC	NCC	Gastric content/ marine litter	Main macro and microscopic findings*	Pathogens and Helminths Detected**	Etiological diagnosis (relevant pathological findings)	COD	Origin	Sub-category	Fishery interaction	Category	Description	Rate	Ref.
30	26617	26/02/2020	Bacoli (Na)	Striped dolphin	F	Juvenile	2	Poor	Absent/ Plastic fragments (intestine)	**Macro: severe generalized lymphadenopathy, associated to hemorrhages (**mesenteric and tracheobronchial lymphnodes); pulmonar congestion; p**arasitic hepatitis; parasitic nodular gastritis**; severe congestion of the duodenal serosa; hyperemic meninges and brain. **Micro:** non-suppurative meningoencephalitis; **reactive lymphadenitis**; disseminated intravascular coagulation (DIC)	**CeMV** (LNs); *P. damselae* (MES ln., intestine); *Crassicauda* spp. (subcutis); *Clistobothrium* spp. (muscle); ***Campula*** spp. (liver); ***P. gastrophilus*** (stomach);	Reactive lymphadenitis associated to *Morbillivirus* infection; unknown origin meningoencephalitis; DIC; multiorganic parasitosis	Natural	Infectious	Viral Parasitic					[61]
31	80795	20/06/2020	Ischia (Na)	Striped dolphin	F	Adult	2	Good	Absent/-	**Macro:** multifocal haemorrhagic, papillomatous-like skin lesions; generalized congestion (liver, spleen, kidney, lung); **severe parasitic nodular gastritis, associated to purulent inflammation area at duodenal level;** mucous endometritis; prescapular lymphadenopathy, associated to haemorrhages. **Micro:** severe pyogranulomatous dermatitis and panniculitis; systemic neutrophilic inflammation; disseminated intravascular coagulation (DIC); parasitic granulomatous pneumonia	*P. damselae* (lung, intestine); *Candida* spp. (skin, vagina); *P. delphini, M. grimaldii* (subcutis, muscle); ***P. gastrophilus*** (stomach)	Severe generalized infectious dermatitis/panniculitis; unknown origin systemic neutrophilic inflammation; DIC; parasitic disease associated to *P. gastrophilus*; parasitic pneumonia	Natural	Infectious	Bacterial Parasitic					[61]
32	93301	16/07/2020	Cetara (Sa)	Striped dolphin	F	Calf	3	Good	Present/-	**Macro:** generalized congestion (liver, spleen, kidney); haemorrhagic pleuropneumonia; sub-occlusive intussusception of the distal part of the intestine; cerebral autolysis. **Micro:** acute pleuritis and	CeMV (kidney); *P. damselae* (liver, kidney, spleen); *Vibrio* spp. (CNS, lung, liver, kidney, spleen)	Haemorrhagic pleuro-pneumoniae; *P. damselae* and *Vibrio* spp systemic infection	Natural	Infectious	Bacterial					[61]

Case ID	IZSM code	Stranding Date	Stranding Location	Species	Sex	Estimated Age Class	DCC	NCC	Gastric content/ marine litter	Main macro and microscopic findings*	Pathogens and Helminths Detected**	Etiological diagnosis (relevant pathological findings)	COD	Origin	Sub-category	Fishery interaction	Category	Description	Rate	Ref.
										bronchopneumonia; hepatic fibrosis										
33	168051	07/11/2020	Anacapri (Na)	Fin whale	F	Juvenile	4	Poor	Absent/-	**Macro: multifocal moderate parasitosis by *Pennella* spp.**; multifocal subcutaneous and muscular edema; generalized autolysis; moderate mesenteric and pelvic lymphadenopathy; severe intestinal parasitisation by Cestoda	CeMV (lung); *P. damselae* (inguinal lymph node, blowhole); ***Pennella*** spp. (subcutis); Cestoda (intestine)	*Morbillivirus* infection	ND							[61]
34	178155	10/12/2020	Ispani (Sa)	Striped dolphin	M	Adult	4	Poor	Absent/-	**Macro:** generalized congestion; **severe haemorrhagic glandular and pyloric gastritis. Micro: severe pyogranulomatous encephalitis associated with mild meningitis; subacute interstitial bronchopneumonia**; **parasitic gastritis**	**CeMV** (CNS, lung, PSC ln, heart, liver; IHC + CNS). ***T. gondii*** (CNS, heart, PSC ln, spleen; IHC+ CNS); *P. damselae* (CNS, lung, spleen); *P. delphini* (blubber); *M. grimaldii* (muscle); ***P. gastrophilus*** (stomach)	Pyogranulomatous meningoencephalitis associated to T. *gondii* systemic infection and *Morbillivirus* systemic infection; *P. damselae* systemic infection; haemorrhagic parasitic gastritis	Natural	Infectious	Viral Bacterial Parasitic					[61,68]
35	186928	29/12/2020	Marina di Camerota (Sa)	Striped dolphin	F	Adult	3	Good	Absent/-	**Macro:** emphysema, haemorrhagic pneumonia and **bronchopneumonia by nematodes**; generalized congestion (spleen, kidney, uterus); hyperaemic meninges, brain and cerebellum. **Micro:** neuronal degeneration, neuronophagia; hyperplastic lymphadenitis; disseminated intravascular coagulation (DIC); chronic gastritis; granulomatous cholangiohepatitis; eosinophilic enteritis; **parasitic** mastitis and **pneumonia**	**CeMV** (CNS, lung, PSC ln); *P. damselae* (CNS, lung, liver, kidney, PSC ln, intestine); *P. delphini* (blubber); *M. grimaldii* (muscle); **Nematoda (lung)**	*Morbillivirus* systemic infection; *P. damselae* systemic infection; DIC; multiorganic parasitosis	Natural	Infectious	Viral Bacterial Parasitic					[61]

Case ID	IZSM code	Stranding Date	Stranding Location	Species	Sex	Estimated Age Class	DCC	NCC	Gastric content/ marine litter	Main macro and microscopic findings*	Pathogens and Helminths Detected**	Etiological diagnosis (relevant pathological findings)	COD	Origin	Sub-category	Fishery interaction	Category	Description	Rate	Ref.
36	10201	15/01/2021	Sorrento (Na)	Fin whale	F	Adult	4	Moderate	Absent/-	**Macro:** scoliosis; large haemorrhagic area in the epigastric region and presence of a well-capsulated abscess in the caudal area; generalized autolysis; extensive destructive phenomena of the vertebral bone tissue associated with osteophytic neoformations from a probable previous chronic inflammatory process attributable to ossifying spondylarthrosis affecting 4 lumbar vertebral bodies, with intervertebral fusion.	CeMV (CNS, lung); *Pennella* spp. (skin)	*Morbillivirus* infection	ND							
37	19918	11/02/2021	Bacoli (Na)	Striped dolphin	M	Adult	2	Good	Scarce/-	**Macro**: pulmonary congestion; congestion and **mild nodular granulomatous glandular gastritis**; hepatic congestion; haemorrhagic enteritis; hyperaemic meninges, brain, cerebellum. **Micro: non-suppurative meningoencephalitis and plexochoroiditis**; disseminated intravascular coagulation (DIC); **congestion and interstitial pneumonia; lymphocytic epicarditis and myocarditis**; chronic parasitic cholangiohepatitis; parasitic gastritis; eosinophylic enteritis; **lymphoplasmacellular nephritis**	***B. ceti*** (spleen); ***Brucella* spp**. (CNS, PSC ln.); ***T. gondii*** (CNS, PSC ln.; IHC + CNS); **CeMV** (lung, heart, liver); ***α-Herpesvirus*** (CNS, kidney); *P. delphini, M. grimaldii* (subcutaneous, muscle); ***P. gastrophilus*** (stomach)	*Morbillivirus* systemic infection*;* DIC; non suppurative meningoencephalitis associated to *T. gondii*, *B. ceti* and *Herpesvirus*	Natural	Infectious	Viral Bacterial Parasitic					[47]
38	22585	18/02/2021	Bacoli (Na)	Striped dolphin	F	Adult	2	Good	Absent/-	**Macro**: haemorrhagic pneumonia; bronchopneumonia by nematodes; mesenteric and prescapular lymphadenomegaly; severe haemorrhagic gastritis; **widespread nodular granulomatous**	**CeMV** (PUL ln, heart, bladder, skin); ***B. ceti*** (CNS); *α-Herpesvirus* (skin); ***ɤ-Herpesvirus*** (lung); *P. delphini, M. grimaldii*	Non suppurative meningoencephalitis associated to *B. ceti*; *Morbillivirus* systemic infection; DIC; bronchointerstitial pneumonia associated to *Herpesvirus* infection; multiorganic parasitosis	Natural	Infectious	Viral Bacterial					[47]]

Case ID	IZSM code	Stranding Date	Stranding Location	Species	Sex	Estimated Age Class	DCC	NCC	Gastric content/ marine litter	Main macro and microscopic findings*	Pathogens and Helminths Detected**	Etiological diagnosis (relevant pathological findings)	COD	Origin	Sub-category	Fishery interaction	Category	Description	Rate	Ref.
										**parasitic gastritis**; generalized congestion (liver, kidney, ovarium); severe muco-haemorrhagic cystitis; hyperaemic meninges, brain, cerebellum. **Micro: severe diffuse non-suppurative meningoencephalitis and moderate plexochoroiditis**; disseminated intravascular coagulation (DIC); acute neutrophilic and histiocytis **bronchointerstitial pneumonia; parasitic gastritis** and enteritis; cholangitis; lymphoplasmacellular splenitis; endometritis	(subcutis, muscle, peritoneal cavity); ***P. gastrophilus*** **(**stomach); Nematoda (lung)									
39	72682	16/07/2021	Pozzuoli (Na)	Striped dolphin	F	Juvenile	4	Poor	Absent/-	**Macro:** generalized autolysis; **nodular granulomatous parasitic gastritis**; haemorrhagic pneumonia; cerebral colliquation **Micro:** generalized thrombosis (heart, lung, intestine); **parasitic gastritis**	CeMV (heart, liver, spleen, kidney) *;* *Brucella* spp (spleen); *S. haemolyticus* (lung); ***P. gastrophilus*** (stomach)	*Morbillivirus* systemic infection; *Brucella* spp infection	Natural	Infectious	Viral					
40	97816	12/10/2021	Procida (Na)	Common bottlenose dolphin	ND	Juvenile	4	ND	Absent/-	**Macro:** cranial fracture (mandibular symphysis and maxillary bone); generalized visceral autolysis; cerebral colliquation	CeMV (lung)	*Morbillivirus* infection	ND							
41	113118	15/11/2021	Ischia (Na)	Common bottlenose dolphin	M	Juvenile	4	Good	Absent/-	**Macro:** generalized autolysis; fibrinous pleuritis; cerebral colliquation	CeMV (skin, kidney); *M. grimaldii* (muscle)	*Morbillivirus* infection; fibrinous pleuritis	ND							
42	114719	18/11/2021	Monte di Procida (Na)	Common bottlenose dolphin	M	Adult	2	Good	Present/-	**Macro: h**emoperitoneum; severe mesenteric lymphadenomegaly; congestion and hepatomegaly; spleen hypertrophy associated to sub-capsular haemorrhages; **nodular granulomatous parasitic gastritis;** intestinal	**CeMV** (CNS, lung, heart, liver, spleen, kidney); *M. grimaldii* (muscle); ***P. gastrophilus*** (stomach)	Lymphocytic cholangitis associated to *Morbillivirus* systemic infection	Natural	Infectious	Viral					

Case ID	IZSM code	Stranding Date	Stranding Location	Species	Sex	Estimated Age Class	DCC	NCC	Gastric content/ marine litter	Main macro and microscopic findings*	Pathogens and Helminths Detected**	Etiological diagnosis (relevant pathological findings)	COD	Origin	Sub-category	Fishery interaction	Category	Description	Rate	Ref.
										intussusception; pulmonary oedema, associated to haemorrhagic pneumonia **Micro: severe lymphocytic cholangitis**; reactive lymphadenitis; lymphoid hyperplasia at splenic level										
43	121502	03/12/2021	Vibonati (Sa)	Striped dolphin	F	Adult	2	Good	Absent/-	**Macro:** prescapular and pulmonar lymphadenopathy (congestion, haemorrhages); **granulomatous parasitic gastritis**; congestion of the gastro-intestinal serosa; multifocal haemorrhages (kidneys; adrenal glands; ascending aorta); pulmonary emphysema. **Micro: non-suppurative meningoencephalitis; splenic lymphoid depletion**; thrombosis, mild lymphocytic perivascular hepatitis, hepatocyte degeneration; subendocardial thrombosis and myocardial degeneration and necrosis; lymphocytic endometritis and ovaritis	**α-*Herpesvirus*** (spleen); ɣ-*Herpesvirus* (LNs); **CeMV** (CNS, heart,spleen, bladder)-, *P. damselae* (lung, liver, kidney, intestine, PUL ln); *Aeromonas* spp. (lung, liver, kidney, intestine; PUL ln); *P. delphini, M. grimaldii* (muscle, subcutaneous); ***P. gastrophilus*** (stomach)	Non suppurative meningoencephalitis and lymphoid depletion associated to *Morbillivirus* systemic infection; *Herpesvirus* infection; *P. damselae* systemic infection; multiorganic parasitosis	Natural	Infectious	Viral Bacterial					
44	122998	06/12/2021	Casalvelino (Sa)	Striped dolphin	F	Adult	3	Good	Absent/-	**Macro**: moderate prescapular and pulmonary lymphadenopathy; pulmonary congestion and emphysema, with broncho-bronchiolar hemorrhages; multifocal haemorrhages (liver, kidney, intestinal serosa, ascending aorta); hyperemic brain. **Micro: moderate multifocal non-suppurative meningoencephalitis and plexochoroiditis**; parasitic cholangitis	CeMV (lung, heart, liver, kidney, bladder, LNs); *A. hydrophila* (CNS, liver, spleen, MES, PSC and PUL LNs); ***B. ceti*** (CNS); *Brucella* spp.(lung); ***Klebsiella oxytoca*** (lung); *P. delphini, M. grimaldii* (subcutis, muscle); ***P. gastrophilus***	Non suppurative meningoencephalitis associated to *B. ceti* infection; *Brucella* spp infection; *Morbillivirus* systemic infection; pyogranulomatous pneumonia associated to *K. oxytoca*	Natural	Infectious	Viral Bacterial					[47]

Case ID	IZSM code	Stranding Date	Stranding Location	Species	Sex	Estimated Age Class	DCC	NCC	Gastric content/ marine litter	Main macro and microscopic findings*	Pathogens and Helminths Detected**	Etiological diagnosis (relevant pathological findings)	COD	Origin	Sub-category	Fishery interaction	Category	Description	Rate	Ref.
										and hepatic lipidosis; mesenteric eosinophilic lymphadenitis; **pyogranulomatous pneumonia;** hypertrophy and degeneration of myocardiocytes; **parasitic gastritis**; granulomatous parasitic enteritis	(stomach); Trematoda (intestine)									
45	83941	21/08/2022	Torre del Greco (Na)	Common bottlenose dolphin	F	Juvenile	2	Good	Absent/-	**Macro:** generalized congestion; severe rectal lymphadenopathy; moderate prescapular and pulmonary lymphadenopathy; hepatomegaly; splenic hypertrophy; pulmonary congestion and oedema, associated to haemorrhagic pneumonia and mild parasitic broncho-pneumonia by nematodes. **Micro:** marked gliosis	*T. gondii* (muscle); *P. damselae, V. alginolyticus* (lung, mediastinal lymph node); *M. grimaldii* (muscle); Nematoda (lung)	Generalized congestion; *T. gondii* infection; cerebral gliosis	ND							
46	96313	03/10/2022	Massa Lubrense (Na)	Striped dolphin	M	Juvenile	4	Poor	Absent/-	**Macro:** generalized autolysis; fibrinous serositis; cerebral colliquation	CeMV (bladder); *P. delphini, M. grimaldii* (muscle, subcutaneous)	*Morbillivirus* infection	ND							

Legend: M, male; F, female; ND, not determined; DD, degenerative disorders; CNS, central nervous system; PSC ln., prescapular lymph node; PUL ln., pulmonary lymph node; MES ln., mesenteric lymph node; LNs, lymph nodes; CeMV, Cetacean Morbillivirus; DIC, disseminated intravascular coagulation: IHC: immunohistochemistry. *: Pathological features associated with infectious agents are shown in bold. **Suggested causative agent of pathological features, based on ancillary test, is shown in bold.
